# Germination Response of MR 219 Rice Variety to Different Exposure Times and Periods of 2450 MHz Microwave Frequency

**DOI:** 10.1155/2013/408026

**Published:** 2013-11-06

**Authors:** Daryush Talei, Alireza Valdiani, Mahmood Maziah, Mohammad Mohsenkhah

**Affiliations:** ^1^Department of Cell and Molecular Biology, Faculty of Biotechnology and Biomolecular Sciences, Universiti Putra Malaysia (UPM), 43400 Serdang, Selangor, Malaysia; ^2^Medicinal Plant Research Centre, Shahed University, Tehran 3319118651, Iran; ^3^Department of Biochemistry, Faculty of Biotechnology and Biomolecular Sciences, Universiti Putra Malaysia (UPM), 43400 Serdang, Selangor, Malaysia; ^4^Department of Electrical, Electronic and System Engineering, Faulty of Engineering, Universiti Putra Malaysia (UPM), 43400 Serdang, Selangor, Malaysia

## Abstract

Germination is a key process in plants' phenological cycles. Accelerating this process could lead to improvment of the seedling growth as well as the cultivation efficiency. To achieve this, the effect of microwave frequency on the germination of rice seeds was examined. The physiological feedbacks of the MR 219 rice variety in terms of seed germination rate (GR), germination percentage (GP), and mean germination time (MGT) were analyzed by exposing its seeds to 2450 MHz of microwave frequency for one, four, seven, and ten hours. It was revealed that exposing the seeds to the microwave frequency for 10 hours resulted in the highest GP. This treatment led to 100% of germination after three days with a mean germination time of 2.1 days. Although the other exposure times of microwave frequency caused the moderate effects on germination with a GP_a3_ ranged from 93% to 98%, they failed to reduce the MGT_a3_. The results showed that ten-hour exposure times of microwave frequency for six days significantly facilitated and improved the germination indices (primary shoot and root length). Therefore, the technique is expected to benefit the improvement of rice seed germination considering its simplicity and efficacy in increasing the germination percentage and rate as well as the primary shoot and root length without causing any environmental toxicity.

## 1. Introduction

Rice (*Oryza sativa* L.) is a staple food source of more than two billion people across the world. Of the total calories consumed globally, 23% are supplied by rice [1]. MR 219 is a Malaysian *indica *rice variety resulted from a cross between MR 137 and MR 151, which was released by the Malaysian Agricultural Research and Development Institute (MARDI), in 2001 [2]. However, the variety is considered a high-yielding rice with a suitable quality in shape and taste, but it is sensitive to environmental changes [3]. The propagation of rice generally occurs through seed and seedling, while germination is a crucial stage in the life cycle of the plant. This process depends on the seed structure and the environmental factors that affect the growth potential of the embryo [4, 5]. The microwave applications have especially been developed in agriculture sector over the past 50 years [6]. Tran [7] was one of the pioneer scientists in this field who realized that the germination of *Acacia longifolia* and *A. Sophorae* seeds could be improved by microwave energy at 2450 MHz. Yet, the case has continuously attracted a great deal of consideration since then. The frequency range of microwaves is between 300 MHz and 300 GHz [8, 9]. Due to their high frequency and short wavelength, they are desirable for communication and radar applications as well. The current advances in technology make works get done easier and cheaper. Simultaneously, requests for applying the microwave energy to every corner of life have mounted. Microwaves have been an interesting research subject even in the current century. Aladjadjiyan [10] reported an increase in germination of *Gleditsia triacanthos*, *Caragana arborescens, Laburnum anagyroides*, and *Robinia pseudoacacia* seeds under microwave electromagnetic treatment. Recently, Ragha et al. [11] proved that a certain level of microwave power and exposure time can improve the germination and seedling vigor of wheat (*Triticum aestivum*), Bengal gram (*Cicer arietinum*), green gram (*Vigna radiata*), and moth bean (*Vigna aconitifolia*), while a further increase in the microwave frequency and power density caused a reduce in seed germination and seedling vigor of the plants. As a matter of fact, rice is one of the highly demanded major crops in the world, providing one-third of the total dietary carbohydrate and supplying 50% to 80% of human's daily calorie intake, especially among the Asian, African, and Latin and South American countries [12]. To date, researches conducted on the propagation of this variety of rice in Malaysia have mostly been focused on its regeneration using tissue culture methods [2, 3, 13]. On the other hand, microwaves took an important place in the electrochemical-based investigations to study the biological behavior of seed germination and enzymatic activities of different plant species [14, 15]. Some unofficial reports comply with the existence of germination problem of the MR 219 rice variety. Due to this issue, the present study was aimed to investigate the effects of 2450 MHz microwave frequency on the germination of its seeds considering the germination percentage and rate, as well as primary shoot and root length. Despite this, the growth quality and yield of the microwave-mediated MR 219 rice seedlings on the paddy fields should be subjected to further investigations in the future as this case has not been scrutinized yet.

## 2. Materials and Methods

### 2.1. Seed

The rice seeds belonging to MR 219 variety were provided by Universiti Putra Malaysia.

### 2.2. Microwave Equipment Specification

A 108.5 mm screw mount dipole antenna (GW.15.2113, 1.58 mW) along with a frequency control oscillator (ZX95-2490 model) including the linear tuning characteristics such as frequency range of 2280–2490 MHz, low phase noise, low pushing and low pulling was exploited in this study. The applied amplifier (Model ZX60-3011) was a 50 Ω, 400 to 3000 MHz low noise. The other features were high dynamic range, wide bandwidth, low noise figure 1.5 dB, 1 dB compression, and medium IP3. The detector (Model ZX47-50+ or ZX47-50LN) was 50 Ω, −50 dBm to +15 dBm, and 10–8000 MHz coaxial power detector's low noise DC output, 20 mVp-p type@ 10 MHz, and high dynamic range, high bandwidth. 

### 2.3. Experimental Technique

The present study was divided to two separate experiments, which was carried out throughout October to December 2012. The first experiment was all about testing different exposure times, which was designed based on Randomized Complete Block Design (RCBD) with five treatments (five different exposure times of microwave frequency) in three replicates to find out the appropriate exposure time of microwave frequency. The treatments were included as C (control), T1 (one hour), T2 (4 hours), T3 (7 hours), and T4 (10 hours). The seeds were sterilized with 10% sodium hypochlorite (NaClO) solution for 10 minutes [16] and thoroughly rinsed with distilled water. Fifty seeds were then soaked in 15 Petri dishes, while Whatman no. 2 filter papers were used as the seedbed in this stage. The Petri dishes were incubated inside the growth chamber (model 6MP6010 Adaptis, Conviron) at 28–30°C and relative humidity 70–85%. The seeds were later subjected to different exposure times of microwave frequency inside a yonolit box with 60 × 60 × 60 cm dimensions. To prevent the waves spreading, the box was wrapped with the aluminium foil. Counting the germinated seeds was started from the third day after soaking. The germination percentages after two (GP_a2_) and three days (GP_a3_) of the experiment were calculated accordingly. The mean germination time (MGT_a3_) was calculated using the formula described by Ellis and Roberts [17]. The germination rate (GR_a3_) was calculated by dividing the germination percentage obtained from each counting to the certain number of the counting day. 

The second experiment was designed according to the first experiment's results, in which the best exposure time (10 hours) was tested further by using different periods. This part of the experiment was also carried out based on Randomized Complete Block Design (RCBD) but, as was mentioned, only using the ten-hour treatment with three replicates.

The treatments were included at different periods of the ten-hour microwave frequency exposure comprising C (control), the first day of soaking (T10), the first two days (T20), the first three days (T30), the first four days (T40), the first five days (T50), the last day only (T01), the last two days (T02), the last three days (T03), the last four days (T04), and the last five days (T05). The seed germination conditions were the same as the first experiment. 

The experiments have been done at 2450 MHz frequency and 1.58 mW energy power [18] for a period of six days. During running the experiment, the seeds were incubated in the same growth chamber at 28–30°C, relative humidity 70–85%, and 12 hours light every day. The seeds were placed in the same yonolit box for 10 hours to be exposed to the microwave frequency. At the end of the experimental period, GP, GR, MGT, and primary shoot and root length (SL and RL) were measured and the treatments were evaluated in terms of the aforementioned seed germination indices. 

### 2.4. Statistical Analysis

Initially, the raw data were tested for normality using the SAS software version 9.2 and the main data were then analyzed using analysis of variance and Duncan's multiple range test in 1% level.

## 3. Results

### 3.1. The Effects of Different Exposure Times of Microwave Frequency on Seed Germination

**Figure 1:**
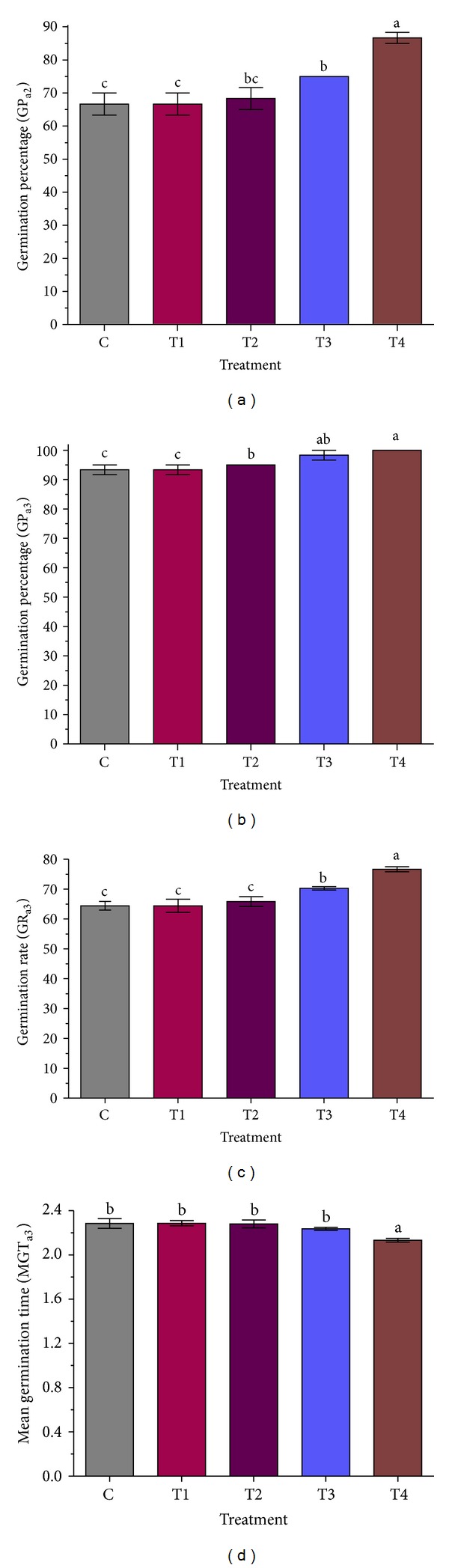
Seed germination of MR 219 rice variety under different times of microwave frequency treatments. (a) Germination percentage after two days of treatment, (b) germination percentage after three days of treatment, (c) germination rate after three days, and (d) mean germination time after three days. Mean values ± SE are from three independent replicates, and values superscripted by different letters are significantly different by Duncan's multiple range test (*P* ≤ 0.01). The treatments were (C) control, (T1) One hour, (T2) four hours, (T3) seven hours, and (T4) ten hours of exposure times of microwave frequency.

Putting different exposure times of microwave frequency to use prior to seed germination significantly accelerated the germination of the rice seeds. Variance analysis of the treatment effects on the measured characteristics (GP_a2_, TGP_a3_, MGT_a3_, GR_a3_, RL_a6_, and SL_a6_) showed that the treatments improved the germination of the seeds, significantly (Table 1). The seeds were successfully germinated with a percentage between 66.7 and 86.7% two days after applying the treatments. The GP_a2_ values showed that the ten-hour microwave frequency was the most effective exposure time to germinate the seeds, so that 66.7% of the seeds germinated after two days (Figure 1(a)), and this percentage was even increased up to 100% after three days (Figure 1(b)). The other exposure times of microwave frequency demonstrated varying effects as reflected by GP_a3_ values ranging from 93 to 98%. 

**Figure 2:**
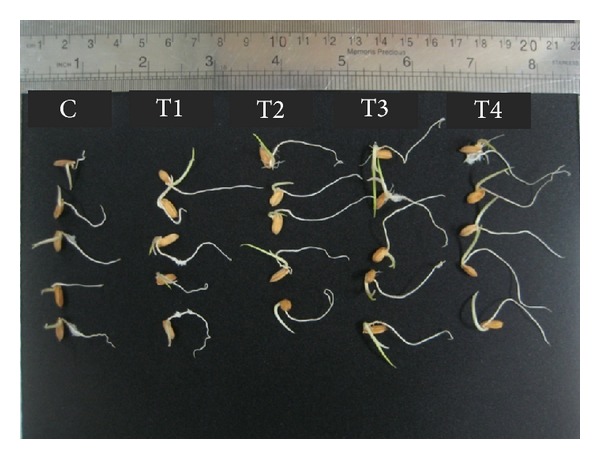
The effects of different exposure times of microwave frequency on primary shoot and root length in MR 219 rice seedlings.

**Figure 3:**
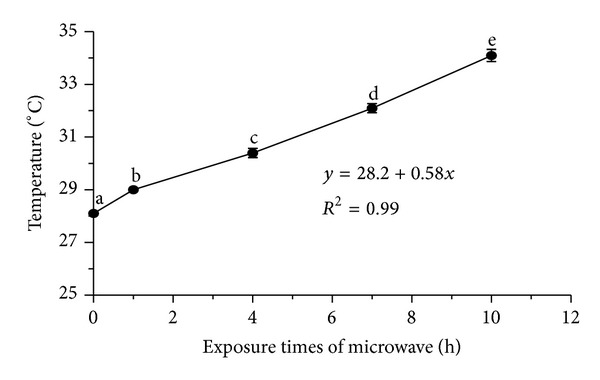
The effect of exposure times of microwave frequency on the water's temperature around the seeds inside the Petri dishes. Vertical bars represent the standard error of mean for three samples, and values superscripted by different letters are significantly different by Duncan's multiple range test (*P* ≤ 0.01).

Exposing the seeds to microwave frequency for ten hours improved the germination rate as shown by the GR_a3_ value (76.7) (Figure 1(c)) and decreased the mean germination time (Figure 1(d)). Furthermore, treating the seedlings with the ten-hour microwave frequency for six days could facilitate and improve the germination indices (primary shoot and root length) in a significant manner (Figure 2). The highest SL and RL at the ten-hour exposure time were 2 and 5.1 cm, respectively (Figure 2). It worth mentioning that thermography examination revealed that increasing the exposure times of microwave frequency led to increase in the temperature of the water inside the Petri dishes around the seeds, significantly (*P* ≤ 0.01). The temperatures of the water inside the Petri dishes after one- and ten-hour exposure times of microwave frequency were 29 to 34.1°C, respectively (Figure 3). 

### 3.2. Effects of Microwave Frequency on Seed Germination of Rice at Different Days

The microwave frequency affected the seed germination of MR 219 rice variety after treating the seeds at different days of soaking with considerable changes in GP, GR, MGT, SL, and RL. Variation due to microwave frequency in all studied traits was highly significant (*P* ≤ 0.01) (Table 2). The studied characteristics were all increased under the microwave frequency effect except for MGT. Comparison of means using Duncan's multiple comparison test indicated two distinct groups in terms of GP_a2_ (*P* ≤ 0.01) (Table 2). As mentioned in Section 2.3, the second experiment of this study was designed in a complimentary way to cover all the possible modes in terms of the experiment's days, so that the first group of the seedlings were treated by the ten-hour microwave frequency from the first day of the experiment by adding the next day to it until the last day of the research (T10–T50) and vice versa, as the same treatment for the second group of the seedlings was applied from the last day of the experiment (T01–T05). In fact, the point of such a strategy was to find out the periodic impact of the ten-hour microwave frequency. This procedure resulted in a surprising outcome, whereas the within and between group differences were found nonsignificant and significant (*P* ≤ 0.01), respectively (Table 2).

**Figure 4:**
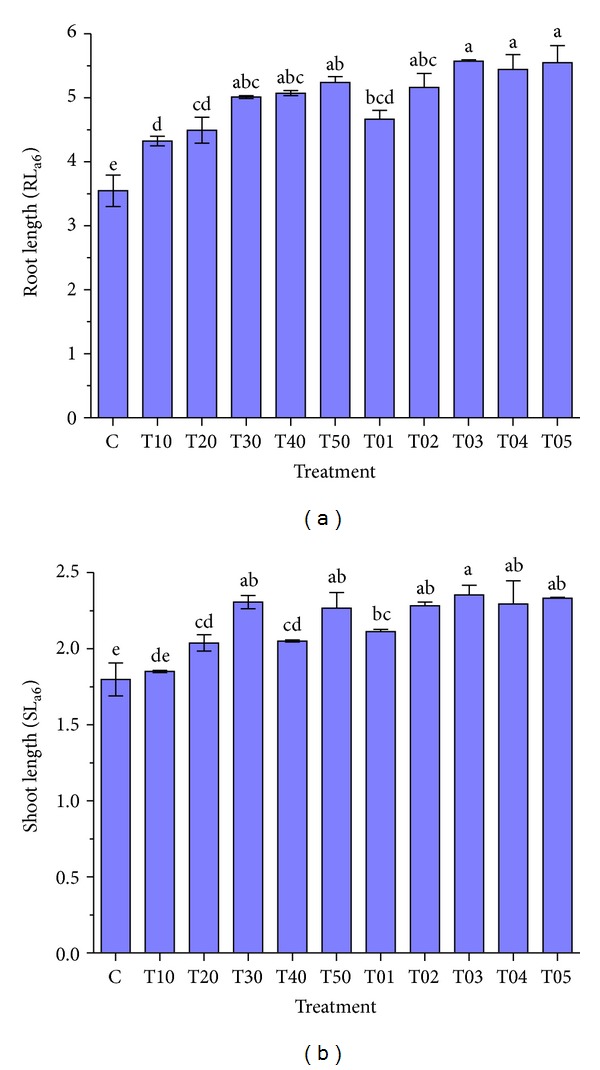
The effect of different periods of microwave frequency on shoot and root length in MR 219 rice variety. (a) Root length after six days and (b) shoot length after six days. Mean values ± SE are from three independent replicates, and values superscripted by different letters are significantly different by Duncan's multiple range test (*P* ≤ 0.01). The treatments were (C) control, (T10) the first day, (T20) the first two days, (T30) the first three days, (T40) the first four days, (T50) the first five days, (T01) the first last day, (T02) the first two days, (T03) the first three days, (T04) the first four days, and (T05) the first five days of ten-hour exposure times.

The mean of GP_a2_ varied significantly between 70% (in control) and 83.3% (in all the treatments excluding the T10 treatment) and this percentage was enhanced up to 100% after three days. The treatments also affected the other studied traits (GP_a3_, MGT_a3_, and GR_a3_), significantly (*P* ≤ 0.01) (Table 2). Interestingly, the same trend of statistical significance was repeated in these traits too, as the within group differences in the treatment groups of T01–T05 and T10–T50 were found nonsignificant, while a significant difference (*P* ≤ 0.01) was observed between these two groups (Table 2). The mean of RL under the T03 treatment varied from 3.6 cm (control) to 5.6 cm (Figure 4(a)), while the SL ranged from 1.8 (control) to 2.4 under the same treatment (T03) (Figure 4(b)). Overall, exposing the seeds to microwave frequency for ten hours in the first two days produced the best results for GP, MGT, and GR (Table 2). Nevertheless, the highest improvement in primary root and shoot length had happened by the ten-hour exposure time for the last three days.

## 4. Discussion

The propagation of rice generally occurs through seeds or seedlings, and seed germination depends on seed structures and environmental factors that affect the growth potential of embryo [5]. In order to investigate the germination of the MR 219 rice variety, a set of critical germination-related indices such as GP, MGT, GR, RL, and SL were evaluated under different exposure times of microwave frequency. All the studied germination indices were affected under different exposure times of microwave frequency. As mentioned in Section 3.1, the thermography examination showed an increase in the water temperature around the seeds of each Petri dish. 

Therefore it can enhance the movement of molecules and improve the growth potential of the embryo. Our results matched up well with the findings of Nelson [19] in cereal grains and Tran [7] in *Acacia longifolia* and *A. sophorae*. Manickavasagan et al. [20] believed that the nonuniform heating pattern of microwaves may stand to reason for formation of hot spots and normal heating zones, while the germination percentage in the normal heating zones could be significantly (*P* ≤ 0.05) higher than the hotspots. In a similar attempt, Rajagopal [6] showed that the microwave frequency facilitated the germination rate and growth indices in wheat, barley, and rye. This may be due to the positive effects of microwave frequency on water absorption and other biochemical processes, as well [19].

According to the results of the present study, exposing the seeds to the ten-hour microwave frequency at the first day of soaking was very important in improving the seed germination percentage and rate. The energy content of the used microwave frequency facilitated the movement of water molecules and perhaps increased the water absorbance by the embryo of the MR 219 rice seeds, and this can be justified by the enhancement in the measured traits such as GP and GR. Unlike these two traits (GP and GR), applying the ten-hour treatment of microwave frequency in the first three days was not efficient enough in improving RL and SL due to the lack of germinated seeds in this stage. In contrast, exposing the germinated seeds during the last three days significantly affected the RL and SL and successfully led to obtain the highest RL and SL values. 

Baskin and Baskin [21] and Basra [22] have stated that germination stage requires a higher amount of temperature compared to the other growth stages in plants, generally. Perhaps this could be another reasonable justification for increasing the germination rate and percentage of long exposure time (the ten-hour treatment). Our results revealed the importance of microwave frequency in improving the seed germination percentage, rate and shoot, root growth. Seemingly, the upshots on shoot and root length are carrying a practical message which matters to those paddy farmers that are using the rice seedlings as the main source of rice propagation in their fields. In other words, the feasibility of this technique defines it as a friendly approach that could be utilized by any local rural cooperatives. However, the overall results suggested the ten-hour treatment of microwave frequency as the best exposure time for germination at the first two days of soaking. Nonetheless, exposing the seedlings to the same treatments for the last three days resulted in better outcomes for shoot and root growth. This investigation proved that treating the seeds with the ten-hour microwave frequency for five days is recommendable for improving the GP, GR, SL, and RL in MR 219 rice variety. The same procedure can be conducted on the other rice varieties which are experiencing a sort of difficulty in their germination stage and potentially the same results can be reproduced by different varieties.

In spite of the interesting results achieved in the current study, we believe that this type of researches should not be limited to the germination stage. For instance, the parallel examination on maize has already revealed the positive impact of microwaves at 945 MHz on germination, growth rate, and absorbance efficiency of photosynthetic pigments [23]. Skulinová et al. [24] have highlighted the impact of the microwave treatment on the biochemical composition of germinated pea (*Pisum sativum* L.). The content of soluble carbohydrates, proteins, and trypsin inhibitor activity was reviewed as criteria of the microwave heating effects in Skulinová's survey [24]. At one glance, the positive impacts of microwave radiation at frequency of 2450 MHz are not deniable [25]. Despite the safety of this method in most of the plant physiology-assisted studies, we are still willing to advise researchers to take the possible risks of the microwave-based examinations into consideration. This recommendation should specifically matter to those who are involved in food-related and biochemical-based researches [24, 26].

## 5. Conclusion

The most effective impacts of microwave energy on the seed germination, shoot and root growth appeared after ten hours of exposure time in the period of five days. However, the mechanisms of the microwave frequencies on germination and plant growth are unknown, but, according to the positive effects of the microwave frequencies on germination indices and shoot and root growth, this technique is expected to benefit the seed germination considering its simplicity and efficiency. Another advantage of this system is improving the seed germination without causing any serious environmental toxicity for producing rice seedlings, which is the central step prior to rice cultivation. Nevertheless, none of these advantages should lead us to a negligence of any probable disadvantage of the technique.

## Figures and Tables

**Table 1:** Variance analysis of the different exposure times of microwave frequency effects on the measured traits in MR 219 rice variety.

Source	df	Mean square					
		GP_a2_	GP_a3_	MGT_a3_	GR_a3_	RL_a6_	SL_a6_
R	2	51.67^ns^	5.00^ns^	0.004^ns^	17.64^ns^	0.002^ns^	0.006^ns^
T	4	219.17**	27.50*	0.013**	82.29**	1.287**	0.053**
Error	8	14.17	5.00	0.002	3.75	0.007	0.004
CV%		19.50	5.21	0.09	5.49	0.16	0.22

**and ^ns^refer to 1% and nonsignificant, respectively. R: block, T: treatment, GP_a2_: germination percentage after two days, GP_a3_: germination percentage after three days, MGT_a3_: mean germination time after three days, GR_a3_: germination rate after three days, Sl_a6_: shoot length after six days, and RL_a6_: root length after six days.

**Table 2:** The effects of microwave frequency on the studied characteristics in MR 219 rice variety.

Treatment	TGP_a2_	TGP_a3_	MGT_a3_	GR_a3_	RL_a6_	SL_a6_
C	70.0 ± 5.8^b^	90.0 ± 0.0^c^	2.2 ± 0.07^a^	65.6 ± 2.4^c^	3.6 ± 0.4^e^	1.8 ± 0.11^f^
T01	70.0 ± 2.9^b^	91.7 ± 1.7^ab^	2.2 ± 0.04^a^	65.6 ± 1.0^c^	4.7 ± 0.1^bcd^	2.1 ± 0.01^bc^
T02	71.7 ± 1.7^b^	91.7 ± 1.7^ab^	2.2 ± 0.00^a^	66.4 ± 1.4^ab^	5.2 ± 0.2^abc^	2.3 ± 0.03^ab^
T03	70.0 ± 2.9^b^	91.7 ± 1.7^ab^	2.2 ± 0.03^a^	65.0 ± 1.4^c^	5.6 ± 0.0^a^	2.4 ± 0.06^a^
T04	73.3 ± 3.3^b^	93.3 ± 1.7^ab^	2.2 ± 0.03^a^	67.8 ± 2.0^ab^	5.4 ± 0.2^a^	2.3 ± 0.15^ab^
T05	75.0 ± 2.9^b^	95.0 ± 0.0^b^	2.2 ± 0.03^a^	69.2 ± 1.4^b^	5.6 ± 0.3^a^	2.3 ± 0.01^ab^
T10	86.7 ± 1.7^a^	93.3 ± 1.7^ab^	2.1 ± 0.02^b^	74.4 ± 1.2^a^	4.3 ± 0.1^d^	1.9 ± 0.01^de^
T20	88.3 ± 1.7^a^	100.0 ± 0.0^a^	2.1 ± 0.02^b^	77.5 ± 0.8^a^	4.5 ± 0.2^cd^	2.0 ± 0.05^cd^
T30	88.3 ± 1.7^a^	100.0 ± 0.0^a^	2.1 ± 0.02^b^	77.5 ± 0.8^a^	5.0 ± 0.0^abc^	2.3 ± 0.04^ab^
T40	88.3 ± 1.7^a^	100.0 ± 0.0^a^	2.1 ± 0.02^b^	77.5 ± 0.8^a^	5.1 ± 0.0^abc^	2.1 ± 0.01^cd^
T50	88.3 ± 1.7^a^	100.0 ± 0.0^a^	2.1 ± 0.02^b^	77.5 ± 0.8^a^	5.2 ± 0.1^ab^	2.3 ± 0.10^ab^

GP_a2_: germination percentage after two days, GP_a3_: germination percentage after three days, MGT_a3_: mean germination time after three days, GR_a3_: germination rate after three days, Sl_a6_: shoot length after six days, and RL_a6_: root length after six days. Different letters indicate a significant difference between the values of pairs of accessions within columns (Duncan's multiple comparison test, *P* ≤ 0.01).
